# The institutional responses to new plant variety protection in China in the context of big data

**DOI:** 10.3389/fpls.2025.1633734

**Published:** 2025-09-19

**Authors:** Fenglei Yu, Xiaochang Liu

**Affiliations:** School of Law, Tianjin University, Tianjin, China

**Keywords:** new plant variety, regulation on the protection of new varieties, data application capacity, data sharing, three-dimensional protection mechanism

## Abstract

Improving the new plant variety protection system is of great significance for China to complete the transformation of modern agriculture. At present, China has initially built up a new plant variety protection system with the Regulation of the People’s Republic of China on the Protection of New Varieties at its core. However, the arrival of the digital era has brought far-reaching impacts and strong impacts on the traditional breeding industry, and at the same time, it also puts forward more stringent requirements on the existing protection system. Based on this, this paper will mainly use the comparative research method and literature analysis method to analyse the systems adopted by other countries for the protection of new plant varieties, and at the same time examine the shortcomings and deficiencies of the current system in China. On this basis, this paper will focus on how to improve the data application capacity, promote data sharing, establish the three-dimensional protection mechanism, and improve the benefit-sharing mechanism, so as to promote the continuous improvement and upgrading of China’s new plant variety protection system.

## 1 Introduction

With the challenges posed by global population growth and climate change, the development of agriculture is facing multiple pressures such as food security, resource constraints, and biodiversity conservation. New plant varieties, as an important outcome of agricultural technological progress, play an irreplaceable role in increasing crop yields, resilience and quality, ensuring food security, and promoting green agricultural development. Therefore, the selection and promotion of new varieties will remain an important factor in the development of modern agriculture in China in the 21st century. The essence of new plant varieties is that human beings, on the basis of mastering the plant genes carried by the propagation material, create and modify varieties with appropriate names, with novelty, specificity, consistency, and stability (Fan, 2020). The cultivation of new plant varieties usually requires a large amount of human resources, material resources, financial resources, as well as long-term scientific research investment. The results of this process embody a high level of intellectual labor and should be fully protected by law. Since the promulgation and implementation of the Regulation of the People’s Republic of China on the Protection of New Varieties (the Regulation on the Protection of New Varieties of Plants below) in 1997, the system of the right to new varieties of plants has been continuously improved, and a multi-level protection system supplemented by the Seed Law of the People’s Republic of China (the Seed Law below) and the Patent Law of the People’s Republic of China (the Patent Law below) has been gradually built up. This system has played a positive role in promoting the technical progress of the seed industry, stimulating variety innovation and optimizing the allocation of germplasm resources.

Plant breeding, as an ancient discipline, was mainly based on experience in the early days, and the selection process was based on natural decisions followed by human selection (Liu et al., 2017). However, with the rapid penetration of emerging technologies such as big data and artificial intelligence into the field of plant breeding, which makes the cultivation, dissemination, and protection of new varieties more complex and diversified, the traditional laws and systems appear to be incapable of dealing with the problems brought by the new technologies (Zhang, 2025). Based on this, this paper will first briefly introduce the application of big data technology in modern breeding. On this basis, it will analyse the strategies of different countries for the protection of new plant varieties and the inadequacy of the current domestic protection methods for new varieties. Finally, based on the background of big data, it will provide some targeted suggestions for improving China’s new plant variety protection system.

## 2 Application of big data technology in modern plant breeding

Modern plant breeding is a modern science based on the collection, preservation, research, and utilization of germplasm resources, extensively utilizing modern technologies and instruments, and combining various breeding methods, with the goal of continuously creating new germplasm resources (Li, 2024). Due to the long growth cycle of plants, significant environmental impacts, and slow data accumulation, the entire process of cultivating new varieties often takes more than several decades. Therefore, plant breeding has traditionally been regarded as a highly empirical and highly cyclical task. However, with the introduction of big data technology, this situation is undergoing a fundamental change.

Firstly, in germplasm resource management, big data technology has achieved a leap in the efficiency of resource utilization. Germplasm resources are important materials for selecting and breeding new types of plants, and they are also important factors affecting agricultural science and technology innovation (Wang and Han, 2022). China has a diverse range of germplasm resources, with a large quantity and wide distribution. However, there are still issues such as being large but not strong (Wang et al., 2023). In recent years, the Institute of Crop Science at the Chinese Academy of Agricultural Sciences has collaborated with the DAMO Academy to develop a smart breeding big data platform for the digital archiving of germplasm resources, aiming to address issues such as high error rates in manual data entry and limited data storage capacity in traditional breeding methods. In addition, the Ministry of Agriculture and Rural Affairs has also promoted the establishment of a national crop germplasm resources sharing service platform and a digital germplasm resources information system, integrating the phenotypic information, origin data and genetic characteristics of major crops such as rice, wheat, maize and soybeans, and realizing the digital management of germplasm resources. Taking maize as an example, the National Maize Improvement Centre establishes a germplasm genealogy database and superiority and inferiority trait association mapping through large-scale molecular marker analysis of existing germplasm materials in the pre-breeding stage. Through big data analysis tools, core material groups of target genotypes such as high yield, drought resistance, disease resistance, etc. can be accurately identified, which significantly improves the efficiency of the primary selection of parents. Overall, big data technology has addressed problems such as ‘information silos’, ‘low efficiency’, and ‘insufficient accuracy’ in crop resource management through comprehensive data management, providing critical support for the conservation, utilization, and innovation of germplasm resources, and contributing to global food security and sustainable agricultural development.

Secondly, in the dimension of breeding technology innovation, the application of big data technology has given rise to a new paradigm of precision breeding. Genomic Selection is one of the most cutting-edge breeding methods, the core of which lies in predicting the breeding value of candidate plants through large-scale genomic data and phenotypic data modelling. Compared with the traditional phenotypic selection, genomic breeding significantly improves the screening efficiency, especially for the complex breeding goals of simultaneous selection of multiple traits. In China, rice is one of the most mature crops in which genomic selection has been applied. The Institute of Crop Science of the Chinese Academy of Agricultural Sciences, using the whole genome data of nearly 10,000 rice lines, has successfully realized two generations of advanced screening in early indica rice breeding by constructing a prediction model of breeding value, which has substantially compressed the breeding cycle. In addition, through the collection of climate, soil, fertilizer, and pest data information, combined with crop growth patterns, to build models to achieve high yield and quality of crops throughout the life cycle of precision agriculture has become a reality (Qi et al., 2019). This way of using big data technology for breeding greatly reduces the number of field trials, improves resource utilization, and realizes the shift from ‘relying on experience to make breeding decisions’ to ‘relying on data and software’ (Liu et al., 2019).

Thirdly, in the variety management aspect, big data technology is reshaping the variety validation system. The registration and validation system of new plant varieties is a key institutional arrangement for the establishment of the rights of plant varieties, which is an important gateway to formally incorporate scientific and technological achievements into the legal protection and market access system (Li, 2012). In China, the validation of new plant varieties needs to go through application and acceptance, variety test, validation and announcement, etc., of which variety test includes regional test, production test and DUS test (Yang et al., 2021). In recent years, Big data technology has been gradually introduced in these processes, promoting the digital and intelligent development of plant variety validation and achieving remarkable results. Specifically, the current DUS testing manual in China mainly covers dozens of morphological characteristics of major crops such as rice, maize and wheat. Through image recognition, digital modelling and other technologies, testing units can structurally and quantitatively input plant traits, colors, lengths, angles, etc., into a standardized electronic description file, achieving rapid comparison and retrieval. The introduction of digital technology has optimized the variety validation system in a number of ways: firstly, it has shortened the testing cycle and improved the utilization of resources. For example, the digital comparison system can complete the similarity check of existing varieties within a few days of application acceptance, avoiding repetitive testing and resource wastage. Secondly, it improves the uniformity of the validation standard. Each testing site shares test manuals, scoring standards and comparison templates through the digital platform, which reduces the differences in human judgement and improves the consistency of the national validation results. Thirdly, it enhances transparency and credibility. The Variety Validation Information Disclosure Platform launched by the Ministry of Agriculture and Rural Affairs has realized the traceability of the whole process of application, testing and deliberation, effectively responding to the question of ‘black box validation’ and enhancing the predictability of the system.

**Figure 1:**
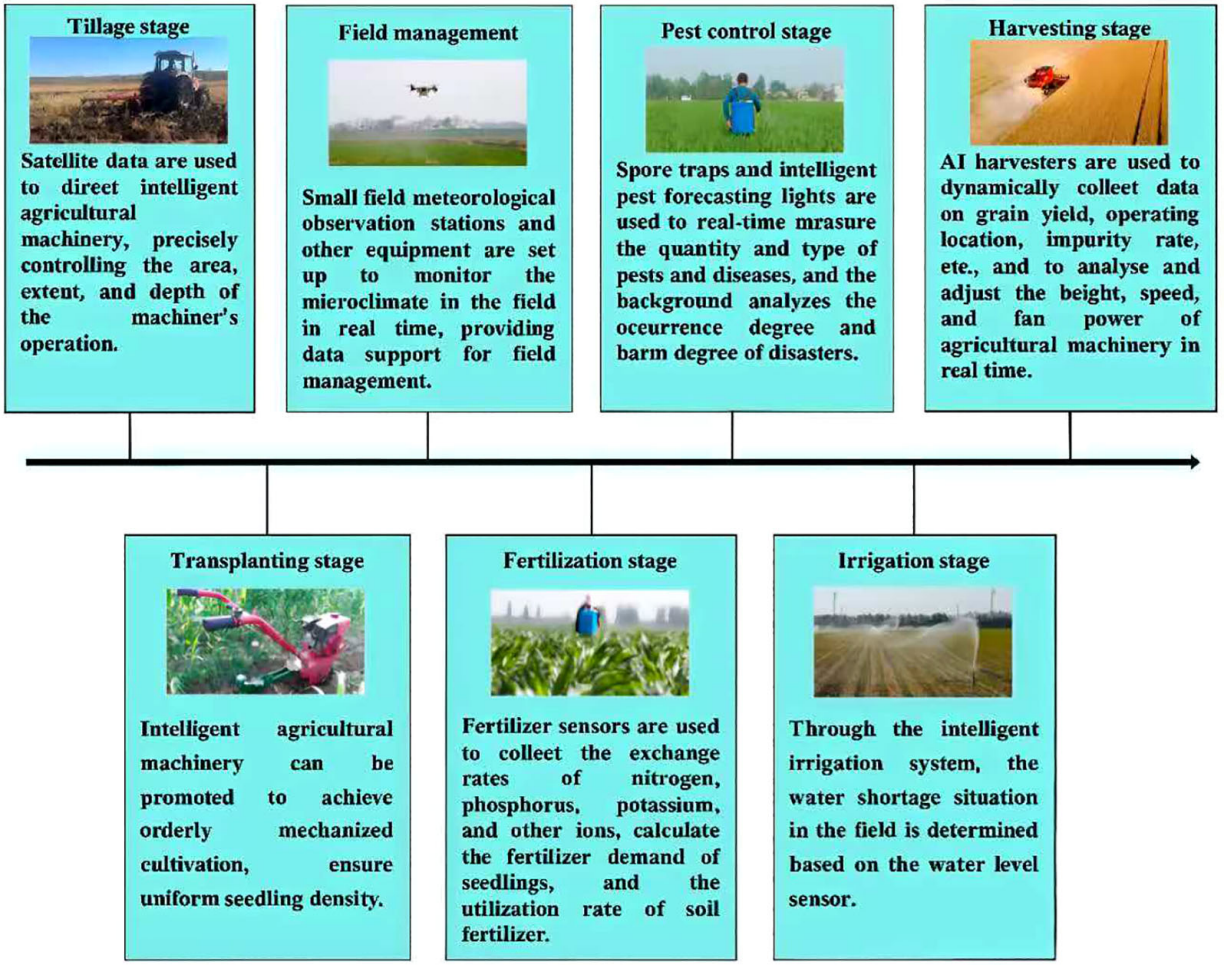
This flowchart illustrates the role of data in each stage of breeding.

Finally, from a practical perspective, the Ministry of Agriculture and Rural Affairs released a series of case studies on the digital transformation of agricultural enterprises in 2025. Zoomlion Smart Agriculture Co., Ltd. began constructing a smart agriculture research and demonstration base in 2016, started exploring and researching digital planting technology for the entire production process of field crops in 2018, and completed the first season of digital rice planting in 2019. In September 2020, the company’s digital rice cultivation technology was approved by expert review, marking the first domestic achievement in digital and standardized rice cultivation. The key technological innovations are illustrated in the following flowchart (**Figure 1**). In 2021, the base was designated as an agricultural and rural informatization demonstration base by the Ministry of Agriculture and Rural Affairs.

## 3 The current status of plant variety protection

### 3.1 Extraterritorial status quo

The protection of new plant varieties is of great significance to guarantee global food security, promote agricultural innovation and maintain ecological diversity. As active promoters of the new plant variety protection system, Europe and the United States have explored different paths for the protection of new plant varieties.

#### 3.1.1 The United States

The United States has constructed a unique composite protection model in the field of intellectual property protection for new plant varieties, which ingeniously integrates patent rights(including plant patents and utility patents) and plant variety rights, in order to cater for the characteristics and innovation methods of different types of new plant varieties. Moreover, breeders do not have to choose only one type of protection. For more valuable varieties, the scope of protection can be increased by stacking rights, providing breeders with comprehensive and flexible protection. The Plant Variety Protection Act (PVPA), the foundational legislation in the protection system, grants breeders exclusive rights to new varieties and applies to both sexually transmitted plants (e.g., those propagated by seed) and tuberous plants (e.g., potatoes). In 2018, the PVPA was revised to include asexually propagated varieties within the scope of variety rights protection. The law is enforced by the Plant Variety Protection Office (PVPO) of the United States Department of Agriculture (USDA). In order to ensure the uniqueness and quality of new varieties, the PVPA requires that varieties applying for protection must satisfy the criteria of novelty, distinctness, uniformity and stability (DUS standard), which is highly consistent with international practice. The PVP certificate holder has the right to exclude others from commericalizing the propagation material of the variety, and at the same time strictly regulates the behavior of other breeders on the variety, thus effectively safeguarding the breeder’s legal rights and interests.

Apart from PVPA, the U.S. Patent Act also provides broader protection for new plant varieties through Plant Patents and Utility Patents. Among them, the US Plant Patent Law’s provision of granting patents for varieties reproduced by asexual reproduction marked the birth of the world’s first national law specifically targeting the protection of new plant varieties (Li et al., 2022). Plant patents are granted to plants that have been cultivated by asexual reproduction, such as by grafting, plugging, or root splitting. The application for a plant patent is subject to a number of strict conditions, namely that the variety must be novel, unique, and capable of being reproduced stably by asexual reproduction. After obtaining a plant patent, the patent holder has the right to prohibit others from propagating the plant by asexual reproduction in the U.S. and to use, offer for sale, sell, and import the propagated plant and its parts, which strongly defends the breeder’s innovations in the field of asexual propagation of plants. It is not difficult to find that the US plant patent system only protects asexual reproduction varieties, but does not protect the widely used sexual reproduction plant varieties in agriculture, nor does it include root and tuber plants. In contrast, the scope of application of utility patents is more extensive, and can be used to protect all kinds of plant-related inventions and creations, including genes, characteristics, methods, plant parts or the whole plant, etc., with no restriction on the kinds of plants and methods of reproduction, even asexually produced plants can be protected by a utility patent, and the objects that can be authorized not only cover plant varieties in a broader sense (including the plant itself and its organs), but also further extend to derivatives directly obtained from them (F1 hybrids, variants, etc.) (Zhong and Wu, 2023). As with other utility patents, utility patents are subject to the patentability requirements of novelty, non-obviousness, and utility. The patent holder has the right to exclude others from making, using, selling, offering for sale, or importing the invention. In summary, the United States has constructed a unique composite protection system through the synergistic operation of plant patents, utility patents and plant variety rights, which has strongly promoted the innovation and development of the U.S. plant breeding industry, and also provided useful reference for the protection of intellectual property rights of new plant varieties around the world.

#### 3.1.2 The European Union

The European Union (EU) has established a solid intellectual property protection barrier for new plant varieties and related technological innovations through the establishment of the Community Plant Variety Right (CPVR) system and the patent system under the European Patent Convention (EPC). Firstly, focusing on the CPVR system, its legal foundation can be traced back to the Regulation on Community Plant Variety Rights (EU-CE2100/94) promulgated in 1994. Taking the 1991 Convention of the International Union for the Protection of New Varieties of Plants (UPOV) as the core framework, this system has constructed a set of normative system covering the whole process of application, examination, protection, and enforcement of plant variety rights. Moreover, the European Union’s plant protection system is an autonomous protection system, independent of the relevant national systems and different from the national laws of member states on variety protection (Luo, 2020). This system is broadly applicable to all types of plant species, including seed-propagated, tuber-propagated, and asexually propagated plants. At the administrative level, the CPVR system is centrally managed by the European Union’s Plant Variety Office (CPVO), which undertakes a number of important duties, including receiving applications, conducting DUS tests, issuing certificates, and maintaining registrations, to ensure that the whole system operates efficiently and in an orderly manner. From the perspective of rights content, CPVR holders enjoy exclusive market control rights, which can prohibit others from unauthorized production, breeding, sale, export or import of breeding materials of protected varieties, and at the same time strictly regulate the development and utilization of derivative varieties, so as to effectively safeguard their own innovations and market interests. However, in order to ensure agricultural production, the act of breeders using the harvested products obtained by planting the breeding materials of protected varieties in the fields for breeding purposes does not constitute infringement. In addition, the CPVR system also has significant internationalization features. By establishing a mutual recognition mechanism for variety test reports with Japan, Australia and other countries, it significantly reduces the administrative costs of cross-border applications and promotes exchanges and co-operation of new plant varieties on a global scale. With the establishment of the unified European Union Plant Variety Protection System, the protection of plant variety innovation through variety rights has become a common choice of European countries.

Turning to the European Patent Convention (EPC), which was signed in 1973 and is administered by the European Patent Office (EPO), the EPC explicitly excludes plant and animal varieties and essentially biological methods of propagation for the production of plants and animals from the scope of patent protection in Article 53(b). When European countries harmonized their domestic patent laws with the EPC, most of them also chose the EPC model, considering that plant varieties or biological methods of producing plants are essentially biological processes, excluding them from protection. However, in the absence of further rules guiding the definition of plant varieties and what constitutes a substantial biological method, this provision has given rise to many controversies, making the issue of patentability of plant bodies or plant varieties particularly complex. For this reason, the EPO has developed important jurisprudential guidance around Article 53(b) of the EPC on whether plant inventions can be protected by patent rights. Since the 1980s, the EPO Technical Appeals Board has, through a series of case law, gradually clarified that genetically modified plants can be used as objects protected by patents (Li, 2013), which has provided a clearer legal basis for the patent protection of plant inventions, and further promoted the innovation and development in the field of plant technology.

#### 3.1.3 India

The protection of new plant varieties is not limited to the protection of breeders’ rights, but often involves issues such as farmers’ rights and genetic resources. The term ‘farmers’ rights’ originated during an internal discussion of the United Nations Food and Agriculture Organization in the late 19th century (Zhang, 2017). At that time, when the participants were debating the conflict of interests between the providers of genetic resources and the providers of technologies, this concept was first proposed. However, the farmers’ rights will inevitably to some extent reduce the scope of protection for new plant varieties’ rights. As a result, this right has been resisted to some extent by certain developed countries. Ultimately, after decades of negotiations, farmers’ rights were recognized by the international community. Under the advocacy of the Food and Agriculture Organization of the United Nations (FAO), the International Treaty on Plant Genetic Resources for Food and Agriculture (ITPGR) was signed in Rome in 2001. Article 9.1 of the Treaty reaffirms the importance of farmers’ rights, stating that the Contracting Parties recognize the significant contributions made and the continued contributions to be made by farmers from local communities, indigenous communities, as well as origin centers and biodiversity centers in various regions of the world to the conservation and development of plant genetic resources for food and agriculture that form the basis of global food and agricultural production. Article 9.2 provides a detailed description of the specific elements of the farmers’ rights: (a) protection of traditional knowledge related to agricultural and food plant genetic resources; (b) the right to participate fairly in sharing the benefits arising from the utilization of agricultural and food plant genetic resources; (c) the right to participate in decision-making at the national level regarding the conservation and sustainable utilization of agricultural and food plant genetic resources (Qiao, 2020).

As a developing country, India has taken the lead in protecting new plant varieties and the rights of farmers. As a major agricultural country, India has abundant domestic plant genetic resources and farmers cultivate diligently using traditional varieties. Against this backdrop, India, based on its own agricultural conditions, drafted the ‘The Protection of Plant Varieties and Farmers’ Rights Act’ (PPVFR) in 1993. After several revisions, it came into effect officially in 2001. As the first domestic legislation to formally introduce the concept of farmers’ rights, PPVFR has set a precedent for the development of domestic legislation on farmers’ rights. To implement this act, the Indian Ministry of Agriculture established the Protection of Plant Varieties and Farmers’ Rights Authority for the purpose of this Act in 2005, which is responsible for managing variety registration, managing farmers’ varieties, managing benefit sharing, collecting and sharing data, etc. In terms of protection scope, the PPVFR’s protection covers extant varieties, farmers’ varieties, and essentially derived varieties. In terms of rights content, farmers are entitled to a wide range of rights, including farmers’ rights, rights of communities, benefit-sharing rights, and the right to claim compensation. In addition, this act has also established relevant systems such as the disclosure system for the sources of plant genetic resources and compensation funds to protect the country’s plant genetic resources (Wei, 2015). In summary, PPVFR provides concrete protection and clearly defines rights and responsibilities. Not only does this establish a protection system, but it also includes specific provisions for punishing infringements, setting an example for developing countries.

### 3.2 Domestic status quo

#### 3.2.1 The composition of the legal system

As an independent type of intellectual property right, the right to new plant varieties occupies an important position in China’s legal system and has a clear and complete institutional basis. To encourage continuous innovation of new varieties, it is necessary to encourage plant breeders to develop superior varieties. The most common system for this purpose is the protection system for new plant varieties (Yu and Chung, 2021). At present, China’s protection of new plant varieties does not rely on a single law, but on a number of laws and regulations to build a rigorous system, including the Regulation on the Protection of New Varieties of Plants, the Seed Law, the Patent Law, and other laws, which constitutes a unique system of agricultural intellectual property rights (Yang et al., 2022). Among them, the Regulation on the Protection of New Varieties of Plants, promulgated by the State Council in 1997, is the core normative document for the protection of new varieties of plants in China, which clearly stipulates the procedures for the application, examination, authorization, movement, and protection of variety rights. At present, China has two variety right validation institutions, the Ministry of Agriculture and Rural Affairs and the National Forestry and Grassland Administration, which are responsible for the management of variety rights of agricultural crops and forest plants respectively, jointly building the first line of defence for the protection of new plant variety rights. Secondly, the revised Seed Law, which came into force on 1 March 2022, has expanded the scope of protection of new plant variety rights, strengthened the liability for damages for infringement, established a system of substantial derivative varieties, intensified the crackdown on fake and inferior seeds, and perfected the infringement compensation system, which provides a solid guarantee for strengthening the awareness of intellectual property rights protection in the seed industry and increasing the protection of new plant varieties in China (Abudukeyoumu et al., 2022). Moreover, although the Patent Law does not directly protect plant varieties in principle, it plays an indispensable role in the protection of plant-related biotechnological achievements. According to Article 25 of the Patent Law, plant and animal varieties are not objects that can be granted patents, but related technological achievements such as transgenic technology, detection methods, and marker-assisted breeding procedures can be applied for as invention patents, thus forming a technical peripheral supplementary protection for plant variety rights. Overall, China has initially formed a legal system for the protection of new plant varieties with the Regulation on the Protection of New Varieties of Plants as the core, supplemented by the Seed Law and the Patent Law, etc., which provides institutional safeguards for coping with innovation in the seed industry.

#### 3.2.2 The Inter-system coordination and conflict

Competition in the international seed industry is, to a large extent, competition in intellectual property rights (Chen et al., 2013). Although the legal protection of new plant variety rights in China has formed a system centered on the Regulation for the Protection of New Varieties of Plants, in actual operation, with the changes of the times and the development of science and technology, the conflict with other intellectual property rights systems are increasingly visible. Especially after big data technology has been deeply integrated into the breeding field, the institutional boundaries between new plant variety rights, patent rights, trade secrets, and data rights have become increasingly blurred, thus leading to extremely complex situations in legal application and rights allocation.

Firstly, there is a boundary conflict between new plant variety rights and patent rights. Article 25 of the Patent Law stipulates that plant and animal varieties shall not be granted patents, and this provision separates the protection of new plant varieties from patent protection at the institutional level, which is a kind of institutional division of the authorization mechanism of the Regulation for the Protection of New Varieties of Plants. However, in actual breeding practice, the situation is far more complicated than the legal provisions described. Especially in the context of the widespread application of transgenic plants, gene editing breeding (Michael et al., 2019), and gene marker-assisted breeding technologies, the plant variety breeding process often integrates a large number of innovative and practical biological technology achievements, and these achievements themselves have the conditions for patent application. Thus, this leads to the phenomenon of ‘dual-track system’ overlap in rights definition. On the one hand, from an overall perspective, new plant varieties are regarded as independent objects and are protected according to the relevant provisions of the variety rights, and this protection mainly focuses on the uniqueness and stability of the varieties; on the other hand, the internal components of new plant varieties, such as the key technical methods used in the breeding process, may be protected by patents, and patent protection emphasizes the innovation and practicality of the technology. However, in practice, due to the fact that in the authorization procedure for new plant variety rights, a strict interpretation is adopted for the authorized plant varieties, while in the authorization procedure for patent rights, a broad interpretation of plant varieties as excluded objects of patent rights is adopted, thus leaving a gap in the protection of intellectual property rights of plant variety-related technologies, resulting in the failure of the object of new plant variety rights and patent rights to be effectively connected (Zhong and Hao, 2023). In addition, there is an essential difference between the patent system and the new plant variety system in terms of disclosure obligations (Guan and Xue, 2017). The patent system emphasizes technical disclosure, while the new plant variety right mainly relies on DUS (distinction, consistency, stability) feature determination, and its disclosure obligation is relatively low. Under the background of big data-assisted examination, there are essential differences between the two systems in terms of technical disclosure standards, data requirements, and examination logic, further increasing the difficulty of system integration.

Secondly, there is a competitive and complementary relationship between the protection of new plant varieties and the protection of trade secrets. Trade secrets, as an interest that does not rely on national registration or public announcement procedures but exists through self-protection methods, are not subject to legal regulations and have strong flexibility (Liu and Zhai, 2025). Breeding units often choose to protect key breeding materials, technical data, genetic genealogy, etc. in the form of trade secrets when new varieties have not yet applied for variety rights, DUS tests have not yet been completed, or for the purpose of circumventing information disclosure requirements. This competitive and complementary relationship becomes more prominent in the context of big data-assisted breeding. Nowadays, relying on data assets such as phenotypic databases, gene editing strategy libraries, and environmental adaptability assessment models has become a reality, and breeding units need to make strategic considerations about whether to choose to apply for new plant variety rights or to maintain the status of a technical secret when faced with these data assets. Some enterprises even tend to bypass the system of new plant variety rights and achieve closed control of germplasm resources through confidentiality agreements and digital platform authority control. This kind of protection can indeed enhance the technical barriers of enterprises in the short term and give them a certain advantage in the market competition. However, from the perspective of the long-term development of the intellectual property system, this kind of behavior may weaken the mobility and sharing of public data resources, resulting in other breeding units being unable to access relevant data information, thus affecting the innovation and development of the entire plant breeding industry. At the same time, it is also detrimental to the transparency and verifiability of plant breeding results, making it difficult to effectively guarantee the quality and reliability of breeding results.

Furthermore, there is an ongoing conflict between plant variety rights and farmers’ rights. Firstly, under the Regulation on the Protection of New Plant Varieties, plant breeders hold exclusive rights to their authorized varieties. Although both the Regulation and the Seed Law explicitly state that farmers’ self-reproduction and use of authorized variety propagation materials do not constitute infringement, the definition of ‘self-reproduction and use’ is vague (Li, 2016). This ambiguity often leads to disputes in practice regarding whether small-scale exchanges are permitted, or whether use beyond the household scope for non-commercial purposes is prohibited. Additionally, many traditional local varieties have been bred and preserved by farmers over generations. Their genetic resources (such as disease resistance or drought tolerance traits) may be used by modern breeders to apply for plant variety rights, but farmers, as holders of traditional knowledge, are often not included as rights holders. The Plant Variety Protection Regulations do not mandate disclosure of the source of genetic resources or farmers’ contributions, thereby posing a risk of ‘resource exploitation.’ Secondly, there is an imbalance in the distribution of benefits between plant variety right holders and farmers. The core of the plant variety rights system is to incentivise breeding innovation by granting exclusive rights to obtain economic returns, while the essence of farmers’ rights is to safeguard basic production rights. The conflicting interests of the two parties directly clash in the commercialization and promotion of breeding. After variety rights protection is implemented, the prices of authorized varieties are dominated by variety right holders (such as seed companies), forcing farmers to purchase expensive seeds and face the challenge of ‘high seed costs.’ Especially in agricultural models dominated by small-scale farmers, seed costs account for a high proportion of production costs. Additionally, China has yet to establish an effective mechanism for interest distribution, leaving farmers’ rights unprotected and exacerbating their resistance to the variety rights system.

Finally, the absence of rules on data property rights can likewise trigger institutional friction. The integration of big data technologies has reshaped the path of plant breeding: from traditional field observations and hybridization trials, gradually shifting to digital breeding processes centered on large-scale genomic analysis, machine learning predictions, and breeding decision systems. Data has transformed from a simple processing object to a fundamental resource (Akoka et al., 2017). This transformation has made data resources more and more important in plant breeding, giving rise to legal concerns about data resources, especially the ownership of key resources such as plant phenotypic databases, germplasm information systems, and varietal mapping libraries. Currently, there is no systematic legal system in China to identify the types of property rights of breeding data and their protection mechanisms. Unclear ownership of data not only affects the open sharing and application efficiency of agricultural breeding data, restricting the further development of agricultural breeding work, but also easily leads to the loss of property rights, making agricultural breeding data lack proper preservation, or even improperly disclosed or incorporated into the control of other people’s rights (Jiang and Chen, 2025). What is more complicated is that data, as an immaterial and infinitely reproducible asset, is difficult to control in the process of its use, which is in structural tension with the traditional intellectual property rights system, which is centered on exclusivity.

In general, there is not only an overlap in rights boundaries, but also a conflict in value objectives between new plant variety rights, patents, trade secrets, and data-sharing mechanisms driven by big data. The new plant variety rights emphasize the protection of the originality of innovative achievements, aiming to encourage breeders to cultivate more unique plant varieties; the patent rights emphasize the protection of the breeding process and its internal components to promote technological innovation and application; trade secrets emphasize information confidentiality to maintain the competitive advantage of enterprises; while the data sharing mechanism driven by big data emphasizes openness and circulation efficiency to promote the development and innovation of the entire industry. Therefore, how to establish a balanced mechanism among these four has become a core challenge that needs to be addressed urgently.

## 4 Institutional optimization of plant variety protection in the digital era

In the current era of rapid development of digital technology, cutting-edge technologies such as big data and artificial intelligence are redefining the paradigm of plant breeding with an unprecedented depth and breadth. This change not only brings unprecedented opportunities for plant breeding, but also poses a systematic challenge to the traditional new plant variety protection system. In order to effectively respond to this challenge, there is an urgent need to build a trinity of institutional frameworks with the premise of improving data application capacity, the core of promoting data sharing, and the bottom line of building a three-dimensional protection system. Through collaborative innovation of legal rules and technological governance, a dynamic balance between seed industry security and technological innovation can be achieved, thereby promoting the sustainable development of the plant breeding industry in the digital age.

### 4.1 The enhancement of data application capacity

The application of big data-related technologies in the field of plant breeding has significantly enhanced the accuracy and efficiency of breeding. However, this advancement also demands higher data application capabilities, which can be improved through enhanced training, technological, and motivation systems.

Firstly, strengthening education and training is conducive to establishing a solid foundation of knowledge for data applications. The key to strengthening breeding technology and seed industry innovation lies in talent, and the degree of synergy in talent policy is an important factor in development (Cheng and Xu, 2020). Therefore, at the level of the education system of colleges and vocational schools, data science, agricultural big data analysis, and other introductory courses should be added, so that students can systematically master the methodology and technology of data collection, collation, and analysis, and apply the theoretical knowledge to the actual problems of breeding. At the same time, the practical teaching link is equally important, establishing an agricultural big data practical teaching base, cooperating with agricultural research institutions and seed enterprises, and providing students with actual breeding data sets, so that they can exercise the ability of variety performance prediction, genetic law analysis and other abilities in practice, and accumulating valuable experience for future career development. For in-service breeders and other subjects, data application capabilities can be enhanced by providing short-term training courses and online learning. Inviting data science experts and senior experts in the breeding field to give lectures, combining theoretical explanations, case analyses, and practical operations, enables in-service users to quickly master practical skills.

Secondly, introducing advanced technologies is beneficial for enhancing the efficiency of data applications. Investment in breeding advanced technologies is a key move to solve the current obstacles to the high-level development of the seed industry (Wan and Li, 2022). The application of technologies such as machine learning and artificial intelligence has brought new breakthroughs in breeding data application. Therefore, relevant algorithm training courses can be conducted, so that breeding personnel and other entities can understand the principles and application scenarios of common algorithms, such as neural networks, which are applied in the classification and prediction of breeding data. Encouraging breeding personnel to apply advanced technologies in actual breeding projects, for example, using machine learning algorithms to mine a large amount of breeding data to discover potential breeding patterns and high-quality gene combinations; using AI technology to construct breeding decision-making models to provide intelligent suggestions for the development of breeding programs; and developing AI-driven DUS testing tools to shorten the cycle of variety validation, improve the accuracy of validation results and provide intellectual property protection of new plant varieties to provide more reliable technical support. In short, through the introduction of advanced tools and technologies, breeders are able to extract valuable information from massive data, improve the scientificity and accuracy of breeders, and promote the development of breeding work in the direction of intelligence and precision.

Thirdly, establishing a data application reward mechanism is favourable to stimulate the intrinsic motivation of data applications. The establishment of an effective data application incentive mechanism is an important guarantee to promote breeders and other subjects to actively improve the ability of data application. Therefore, scientific research awards related to data application can be set up, such as the ‘Agricultural Big Data Application Innovation Award’, to give recognition and rewards to breeders who have achieved outstanding results in data collection, analysis, and application. Relevant policies should be introduced to support the evaluation of titles, scientific research projects and evaluation of results (Zhou et al., 2023), giving priority to projects with innovative points of data application, and providing more financial support and policy concessions for the development of data-driven breeding research projects, so as to guide breeders to actively explore the application of data in breeding, and to promote the development of innovation in the field of breeding.

### 4.2 The promotion of data sharing

Big data breeding relies on the integration of genomic, phenotypic, and environmental data, but there is a problem of ‘data silos’ in China, where data from research institutes, enterprises, and government departments are stored in a fragmented manner and there is a lack of sharing mechanisms (Li, 2025). This not only limits the efficiency of breeding, but also may lead to intellectual property disputes, such as the unauthorized use of other people’s data. In order to solve the ‘data silo’ problem, there is an urgent need to build an open and transparent sharing mechanism.

Firstly, the establishment of a national agricultural data-sharing platform is the core measure to solve the problem of ‘data silos’. The platform should integrate genomic, phenotypic, and environmental data to form a standardized resource base. Specifically, the Ministry of Agriculture and Rural Affairs or the National Intellectual Property Administration can take the lead, jointly with relevant research institutions such as agricultural academies, to establish a ‘National Agricultural Data Sharing Center’, providing a unified data access entry point. In addition, in the current era of big data, the rapid operation and iteration of algorithms bring massive data accumulation and real-time data updates (Liu, 2023). Therefore, the platform should also establish a data update mechanism to ensure the timeliness and accuracy of data. In addition, the platform should also adopt advanced data encryption technology and access control strategies to prevent data leakage and misuse.

Secondly, the introduction of advanced technologies such as blockchain to track data usage can solve the concerns of data sharing subjects. Blockchain technology, with its tamper-proof characteristics, can accurately record key information such as genome sequences and test data of varieties, effectively preventing data tampering or theft, and is conducive to promoting data sharing (Lei and Liao, 2024). Therefore, to ensure data transparency and traceability, a blockchain-based variety data tracking system can be actively developed. For example, in the process of variety validation, the origin of varieties, the breeding process, and relevant test data can be clearly traced through blockchain technology, providing strong evidence support for intellectual property protection.

Thirdly, the formulation of scientific and reasonable breeding data-sharing rules is crucial for regulating data use behavior and guaranteeing orderly sharing. Relevant provisions on ‘breeding data’ can be added to the Regulations on New Varieties of Plants to improve the provisions on data ownership. On the one hand, the scope and methods of data sharing should be clearly defined. This can be done through clauses such as fair use and legal licensing, which specify which data can be shared, as well as the specific scope and methods of sharing, and establish a convenient data acquisition mechanism (Kerber, 2016). For data involving national security, commercial secrets, and personal privacy, the scope of sharing shall be strictly limited.

On the other hand, while promoting the sharing and use of breeding data, it is also necessary to regulate data usage behaviour. Especially when data involves farmers’ rights, it may also involve sensitive information such as individual farmers’ privacy. Therefore, for the use and sharing of such data, data users should be required to anonymize the data before use or sharing to ensure that such data is usable but not viewable. For data usage or sharing that infringes on privacy, the relevant parties should bear corresponding liability for infringement. Additionally, the use and sharing of data must comply with laws, regulations, and ethical standards. Data must not be used for illegal purposes or to infringe upon the rights of the original data owners. For example, data sources must be properly cited, and data must not be altered without authorization. Any violations of these rules must be promptly addressed and penalized to ensure that breeding data sharing operates in a legal and compliant track.

### 4.3 The establishment of a three-dimensional protection mechanism

Providing intellectual property protection for plant breeding innovation is the inevitable result of the development of agricultural breeding technology and the commercialization of agriculture (Li, 2020). The protection of new plant varieties urgently requires the establishment of a three-dimensional protection system centered on breeders’ rights, supplemented by patent rights and trade secrets. Under the dual-track framework of the International Union for the Protection of New Varieties of Plants (UPOV) Convention and the TRIPS Agreement(Agreement on Trade-Related Aspects of Intellectual Property Rights), the protection of new varieties of plants presents a unique system of complementarity. The protection of new plant varieties (in accordance with the Regulation on the Protection of New Plant Varieties) mainly focuses on the plant varieties that meet the DUS criteria (specificity, uniformity, and stability), and its core lies in the control of the breeding materials; while patent rights protection is applicable to non-traditional breeding methods (such as gene editing technology), and its protection scope extends to the technical scheme itself, but it must meet the requirements of novelty, creativity, and practicality; trade secret protection (based on the ‘Anti-Unfair Competition Law’) provides supplementary protection for the undisclosed breeding technology information, forming a complete protection chain of ‘process - result’ with the previous two rights. This three-dimensional protection framework achieves comprehensive coverage of the ‘variety - method - data’ aspects of new plant varieties: plant variety rights protect the expressional characteristics of the varieties, patent rights protect the creative characteristics of the varieties, while trade secrets can protect the process-related information of breeding, and these three complement each other and enhance each other’s effectiveness.

In terms of the interface between specific systems, a systematic coordination mechanism needs to be established to resolve potential conflicts. Firstly, in the coordination between new plant variety rights and patent rights, a ‘demarcation-linkage’ mechanism should be established. On one hand, a clear ‘list of patentable breeding methods’ should be formulated, forming a ‘method-variety’ vertical division with the protection of plant variety rights. For example, traditional breeding methods, such as hybrid optimization techniques, can be excluded from patent protection, and applicants can be guided to choose variety right protection. On the other hand, for the issue of overlapping rights, the patent system should further clarify the ‘plant varieties’ that cannot be granted patents. Through the issuance of judicial interpretations or guidance cases, the definition of relevant patent rights can be made clearer and more operational, providing legal protection for breeding technology innovation and enabling breeding innovation achievements to be included in the new variety intellectual property protection system as much as possible (Ran, 2024). Secondly, in terms of trade secret protection, it is necessary to establish an embedded protection mechanism. For the key data in the breeding process, a ‘double record system’ should be established, making the biological characteristic data necessary for DUS tests public, while the core breeding parameters can be subject to trade secret protection. At the same time, a breeding database system with encryption function should be developed, and the data can be ‘partially disclosed’ through blockchain technology. In the mechanism of rights protection, it is allowed to claim trade secret infringement incidentally in variety right infringement litigation. In summary, through refined institutional design, not only can each intellectual property system maximize its effectiveness, but also conflicts of rights can be effectively avoided, providing comprehensive legal protection for new plant varieties.

### 4.4 The Innovation of benefit-sharing mechanism

Firstly, the current legal ambiguity regarding the scope of ‘self-retained seeds’ and the definition of traditional variety rights is at the core of the contradiction, necessitating legislative clarification of rights and obligations. Specific measures include: revising the Regulation on the Protection of New Varieties of Plants’ to clarify the boundaries of authorized varieties for ‘self-propagation and self-use,’ balancing traditional practices with variety rights protection; establishing standards and a registration platform for traditional varieties, granting farmers ‘the right to informed consent,’ and ‘the right to share in benefits’ (Liu and Zhang, 2016), legally acknowledging farmers’ contributions to the breeding and preservation of traditional varieties, and balancing the expansion of intellectual property rights with the protection of traditional rights.

Secondly, regarding the issue of resource exploitation and imbalanced distribution of benefits, the risk stems from breeders’ uncompensated use of genetic resources preserved by farmers. This must be addressed through mandatory disclosure, compensation mechanisms, and collaborative models to curb ‘resource exploitation’ and achieve balanced distribution of benefits. Specific measures include: adding a ‘disclosure of genetic resource origin’ requirement in variety rights applications, mandating breeders to specify the sources of traditional varieties, wild resources, or farmer-selected materials used in new variety development. Applications that fail to disclose this information or provide false information will not be authorized; Establishing a ‘National Agricultural Genetic Resources Compensation Fund’ funded jointly by the government, enterprises, and research institutions, which would allocate a certain percentage of the commercial benefits from new varieties to compensate traditional variety providers (such as registered farmers or communities), with priority given to supporting resource conservation and the preservation of traditional knowledge; Encourage ‘enterprise-farmer’ cooperative breeding through resource sharing and shared rights (such as shared variety rights and proportional revenue distribution) to establish long-term cooperative relationships, thereby avoiding the contradiction of ‘free acquisition of resources and high-priced seed sales.’

## 5 Conclusion

In the digital age, the protection of new plant variety rights has become increasingly important and complex. With the rapid development of digital technology, the traditional way of intellectual property protection is facing unprecedented challenges and opportunities. By analyzing the impact of big data technology on each stage of breeding, it is revealed that digitization has a profound influence on the protection of new plant varieties. In order to effectively respond to the challenges posed by the application of big data, it is urgent to construct a trinity institutional framework. The framework is based on the premise of improving data application capacity, promoting data sharing as the core, and building a three-dimensional protection system as the bottom line. In terms of enhancing data application capabilities, efforts can be made from multiple dimensions: strengthening education and training to lay a solid knowledge foundation for relevant personnel; actively introducing advanced technologies to significantly improve the efficiency of data application; establishing a data application reward mechanism to fully stimulate the internal motivation for data application. In terms of promoting data sharing, practical measures need to be taken: building a national-level data sharing platform to provide a unified and convenient data access entry point for all parties; introducing cutting-edge technologies such as blockchain to eliminate the concerns of sharing entities; formulating scientific and reasonable data sharing rules to regulate data usage behavior. In terms of the construction of the three-dimensional protection mechanism, it is necessary to achieve comprehensive coverage of ‘varieties - methods - data’. Plant variety rights focus on protecting the expressive features of the variety, patent rights focus on protecting the creative features of the variety, and trade secrets focus on protecting the process information of breeding. The three complement each other and work together to jointly build a solid defense line for the protection of new plant varieties. Finally, through the formulation of legislation and corresponding rules, the mechanism for sharing benefits will be continuously improved, thereby activating the value distribution hub throughout the entire chain of seed industry innovation.

## Data Availability

The original contributions presented in the study are included in the article/supplementary material. Further inquiries can be directed to the corresponding author.
